# Magneto-Impedance Sensor Driven by 400 MHz Logarithmic Amplifier

**DOI:** 10.3390/mi10060355

**Published:** 2019-05-29

**Authors:** Tomoo Nakai

**Affiliations:** Industrial Technology Institute, Miyagi Prefectural Government, Sendai 981-3206, Japan; nakai-to693@pref.miyagi.lg.jp; Tel.: +81-22-377-8700

**Keywords:** magneto-impedance sensor, thin-film, high frequency, logarithmic amplifier, nondestructive inspection

## Abstract

A thin-film magnetic field sensor is useful for detecting foreign matters and nanoparticles included in industrial and medical products. It can detect a small piece of tool steel chipping or breakage inside the products nondestructively. An inspection of all items in the manufacturing process is desirable for the smart manufacturing system. This report provides an impressive candidate for realizing this target. A thin-film magneto-impedance sensor has an extremely high sensitivity, especially, it is driven by alternatiing current (AC) around 500 MHz. For driving the sensor in such high frequency, a special circuit is needed for detecting an impedance variation of the sensor. In this paper, a logarithmic amplifier for detecting a signal level of 400 MHz output of the sensor is proposed. The logarithmic amplifier is almost 5 mm × 5 mm size small IC-chip which is widely used in wireless devices such as cell phones for detecting high-frequency signal level. The merit of the amplifier is that it can translate hundreds of MHz signal to a direct current (DC) voltage signal which is proportional to the radio frequency (RF)signal by only one IC-chip, so that the combination of a chip Voltage Controlled Oscillator (VCO), a magneto-impedance (MI) sensor and the logarithmic amplifier can compose a simple sensor driving circuit.

## 1. Introduction

A thin-film magneto-impedance sensor [1,2,3,4,5,6] is useful for detecting magnetic materials nondestructively. The sensor has high sensitivity and also has tolerance of normal magnetic field because of its demagnetizing force in the thickness direction. Our previous report showed that this sensor has sensitivity of 1.7 × 10^−9^ T at 300 Hz inside the normal field of 0.1 T [7]. By using this sensor, a single magnetic particle with 65 μm diameter could be detected in the distance 0.5 mm above the sensor plane. In this measurement, the output signal was over 6 V out of the measurement of the single 65 μm particle with a low remanence property. The point is that it was detected with subjecting a static normal field of 0.1 T around a measurement area including the thin-film sensor. Based on the previous work, we are trying to extend the application of this sensor system. The aim of this study is developing a method of nondestructive detection of magnetic small bodies such as foreign matters included in industrial products and also detection of a density of magnetic nanoparticles in a drug solution. In the case of the industrial application, it can detect a small piece of tool steel chipping or breakage inside the products nondestructively. An inspection of all items in the manufacturing process is desirable for the smart manufacturing system. In order to apply an actual manufacturing process a system without magnetic shielding is desirable due to its low cost and small installation space of equipment. An environmental noise in a factory which overwhelms the detected signal can be solved by making the detection signal stronger using a stronger normal magnetic field. The thinner the sensor thickness the more tolerance appears against strong normal field, because of the demagnetizing field. For driving such thin sensor, high frequency is essential for the skin-effect of magneto-impedance. For the measurement system which applied for a conveyer in manufacturing process and medical application the signal frequency range is expected to be from DC to several Hz due to the movement speed of products or scanning speed of measurement probe. This paper provides an impressive candidate for realizing this target. A Co_85_Nb_12_Zr_3_ thin-film magneto-impedance sensor has an extremely high sensitivity, especially, it is driven by AC current around 500 MHz [8]. For driving the sensor in such high frequency, a special circuit is needed for detecting an impedance variation of the sensor. Previous works which report high frequency magneto-impedance [9,10,11] used impedance-analyzer, network-analuzer and signal-analyzer for detection. In this paper, an application of a logarithmic amplifier for detecting a level of the sensor signal at 400 MHz, which is proportional to the sensor impedance, is proposed. The logarithmic amplifier is almost 5 mm × 5 mm size small IC-chip which is widely used in wireless devices such as cell phones for detecting high-frequency signal level. The merit of the amplifier is that it can transfer hundreds of MHz signal to a DC voltage signal which is proportional to the RF signal by only one IC-chip, so that the combination of a chip Voltage Controlled Oscillator (VCO), a MI sensor and the logarithmic amplifier can compose a simple sensor driving circuit. By using the logarithmic amplifier, the output of the sensor circuit suited for a frequency range from DC to several Hz. The sensing circuit has a configuration of differential input for the purpose of getting high sensitivity. One of the inputs is a sensor signal and the other is a reference signal which has the same phase and the amplitude as the one where an external magnetic field is a certain reference value. The output frequency bandwidth of the logarithmic amplifier was ranging from DC to 20 kHz. But in this study the output frequency range was limited in which a signal of measured object carried on a conveyor. This frequency was under several Hz, and the system will be designed without using a magnetic field shield structure. The most sensitive frequency range of our sensor element was around 500 MHz, due to the element sensitivity dZ/dH marks a maximum around 500 MHz, here Z is element impedance and H is external field in the sensing direction. Whereas the circuit in this study was 400 MHz. The reason is a selection of devices which are commercially accessible. If a suitable device would be getting accessible the 500 MHz driving circuit can be made using the same design rule as this paper.

## 2. Experimental Procedures and Results

### 2.1. Sensor Element and Driving Circuit

**Figure 1:**
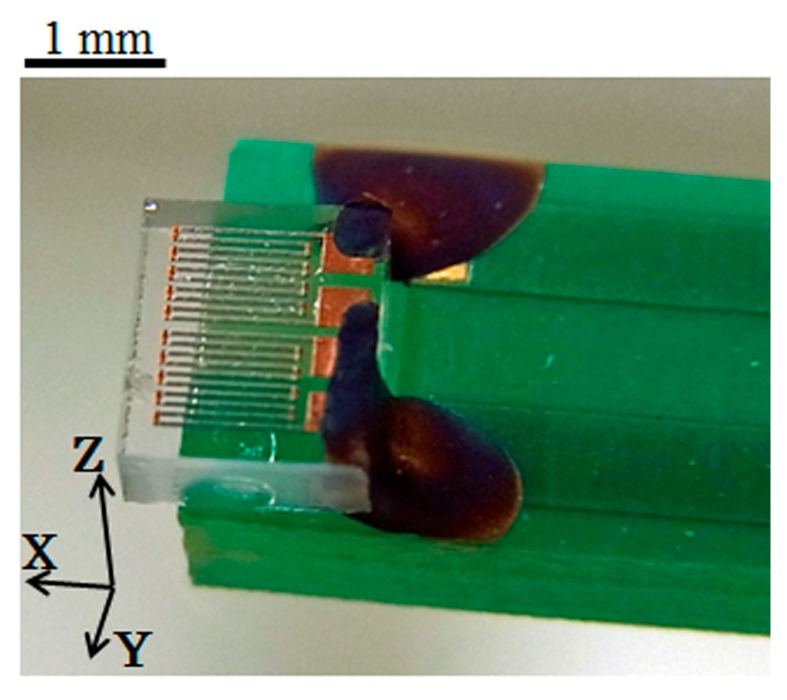
View of the sensor element.

Figure 1 shows the view of sensor element. The element was fabricated by a thin-film process. An amorphous Co_85_Nb_12_Zr_3_ film was RF-sputter deposited onto a soda glass substrate and then micro-fabricated into rectangular elements by a lift-off process. The element was 1000 μm length, 50 μm wide, 2.1 μm thick. The tens of elements are aligned in a parallel configuration and connected by Cu thin-film strips to form a meander pattern. A magnetic field was applied while the RF-sputter deposition for the purpose of inducing uniaxial magnetic anisotropy. The direction of the magnetic anisotropy in this study was in the width direction, Y, so to say short side direction of the element strip. It is induced by the direction of the magnetic field while sputtering. The sensor element was mounted on printed circuit board (PCB) and electrically connected by a conductive silver paste.

**Figure 2:**
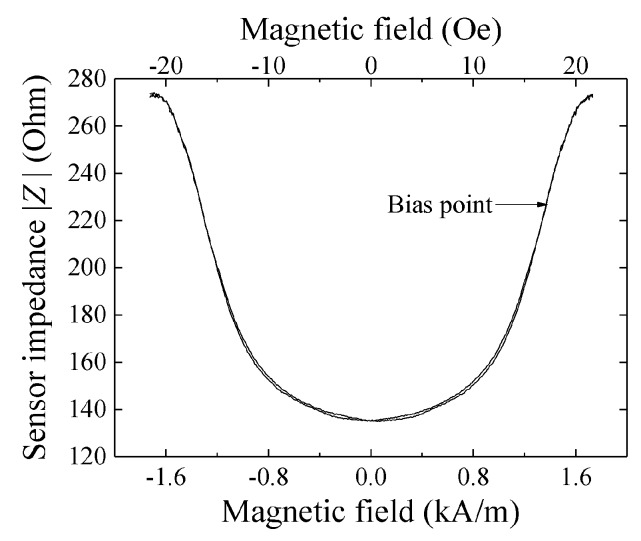
Typical example of variation of impedance of the sensor.

A typical example of variation of impedance of the sensor itself without a PCB as a function of external field is shown in Figure 2. In this figure, the external field was applied in the in-plane length direction of the sensor strip, X-direction, which is the sensing direction. This impedance variation was obtained by S11 measurement of a network-analyzer using high-frequency probe for electric connection with the electrode pads of sensor element. The result shows a parabolic variation, which is typical for a magneto-impedance sensor having magnetic anisotropy in the width direction. The minimum impedance, |Z|=138 Ω at 0 kA/m (0 Oe), and the maximum was 275 Ω around ±1.76 kA/m (±22 Oe). In this figure, a bias point is shown, on which this sensor was operated.

**Figure 3:**
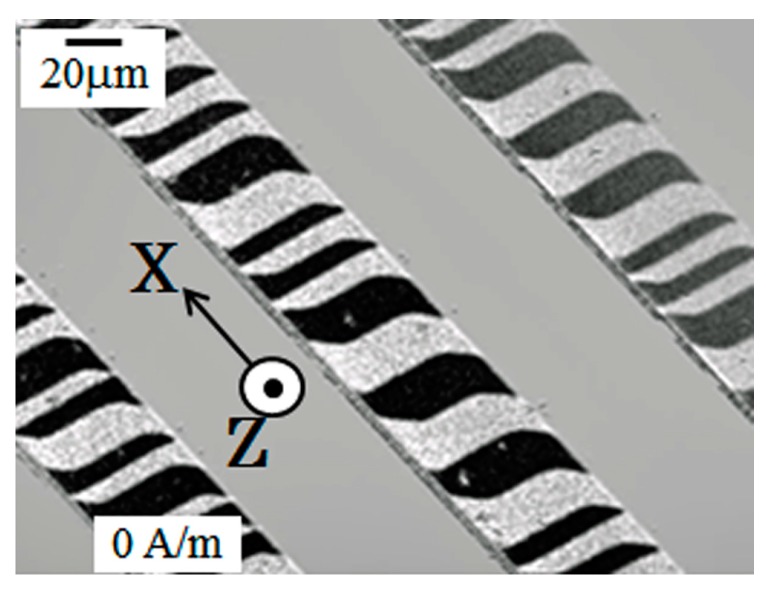
Magnetic domain of the sensor element observed by Kerr microscope.

Figure 3 shows the magnetic domain of the sensor element observed by Kerr microscope (BH-762PI-MAE, NEOARK Corporation, Tokyo, Japan). The magnetic domain forms contiguous regions of magnetic momentum with anti-parallel direction. This picture shows a magnetic domain when an external magnetic field was zero. As increasing or decreasing the external field applied along the length direction, X, which is the sensing direction of sensor, the width of the contiguous domain regions changes and finally the domain would be a single domain with a momentum directing the same as the external field direction. In this element, the magnetic momentum in each region vibrates by the effect of the high-frequency current running through the element, and the impedance of the element changes as the magnetic domain changes. In another word, the magneto-impedance effect comes from the change of the condition of the momentum vibration caused by the change of both the direction and the distribution of the momentum in the thin-film element having an in-plane uniaxial magnetic anisotropy.

**Figure 4:**
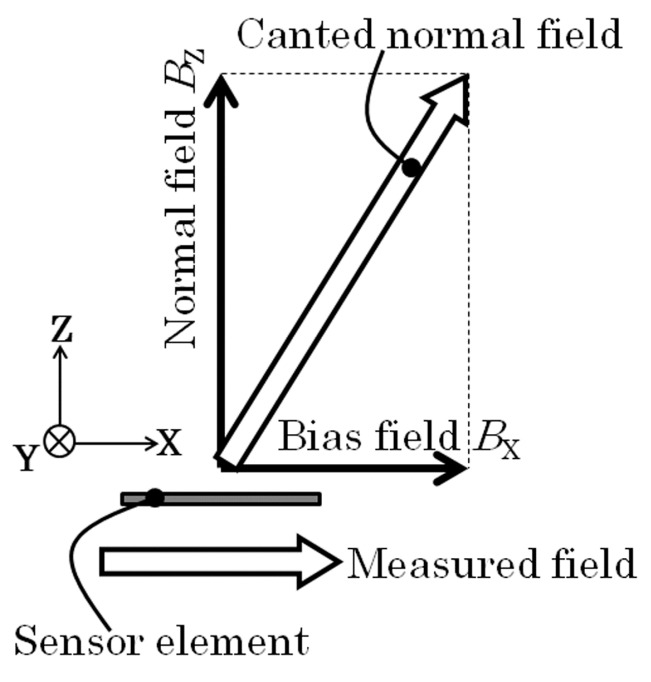
Schematic of magnetic field when the sensor is operated.

Figure 4 shows a schematic of magnetic field when the sensor is operated. The sensor needs a biasing field for operation in the X direction, it is about 1.4 kA/m (17.5 Oe) as shown in Figure 2. The sensing magnetic field is also in X direction. Therefore, a constant bias field is essential for this sensor system. 

**Figure 5:**
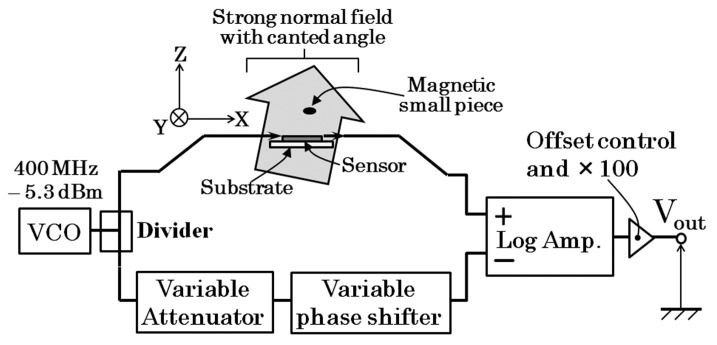
Proposed driving circuit of the thin-film sensor.

Figure 5 shows the proposed driving circuit of the thin-film sensor. A 400 MHz alternating signal at −5.3 dBm was generated by a voltage-controlled oscillator. The signal was divided into two and one was introduced to the sensor element and the other was introduced to a series of RF control devices. As shown in the figure the latter is a series of variable attenuator and a variable phase shifter. Both of them were electrically voltage-controlled ones. The RF signal going through the latter way of circuit branch was controlled in the same phase and amplitude as the one going through the sensor when an external magnetic field is a certain reference value. Each signal was inputted to a logarithmic amplifier as a pair of differential inputs. The logarithmic amplifier detects 400 MHz signal into a voltage signal logarithmically proportional to the signal level. A final output of the circuit was processed with an offset compensation and an amplifying 100 times. The baseline stability of the output is satisfactory even its high-sensitivity. 

### 2.2. Results of Experiment

**Figure 6:**
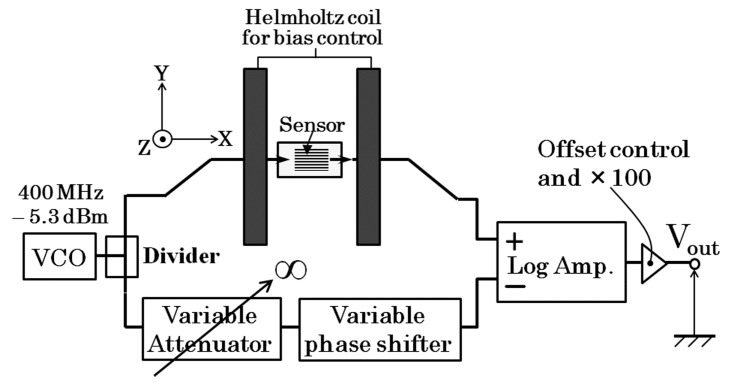
Method of confirmation of certainty of the output of sensor system.

At first, a certainty of the output of this sensor system is confirmed. The system was composed of the sensor on PCB (Figure 1) and driving circuit (Figure 6). It is confirmed by measuring the output signal of the system caused by the change of the element impedance. This measurement was carried out by applying magnetic field in the X direction to the sensor on PCB with driving it by the circuit. The variation of element impedance without PCB is previously shown in Figure 2. The driving circuit was the same as Figure 5 but the attenuator was set to be maximum attenuation. This setting makes an input of the ‘–‘ terminal of the logarithmic amplifier to be almost zero, then the output was expected to be logarithmically proportional to the sensor impedance. 

**Figure 7:**
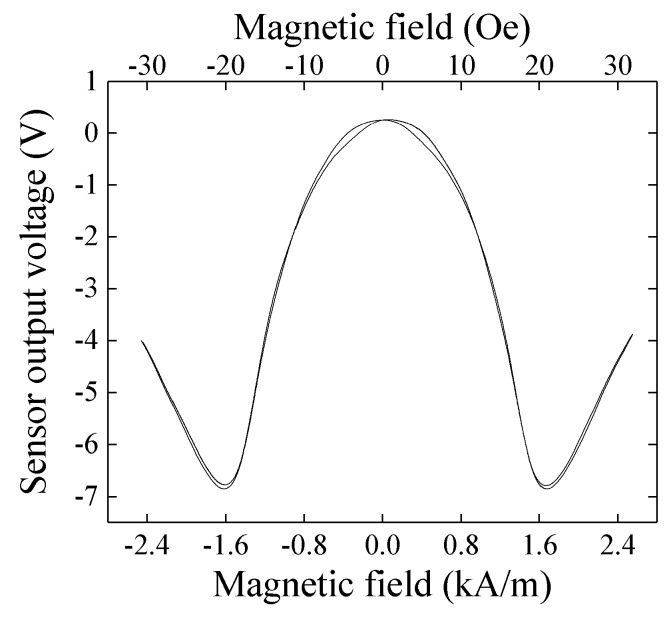
Result of sensor impedance measurement by the circuit.

Figure 7 shows a result of measurement. The external magnetic field was applied in X direction.

In this measurement, the normal magnetic field was in a room condition of Japanese north-east region. The output voltage was ranging from −7 V to +0.2 V and it was in good agreement with the impedance profile of the sensor element. In our measurement the baseline level was an arbitrary one because a level of offset compensation in the final circuit stage was arbitrary. The profile of sensor output (Figure 7) was a vertical inversion of the element impedance (Figure 2). The reason is that the RF level come out from sensor element is inversely proportional to its impedance.

**Figure 8:**
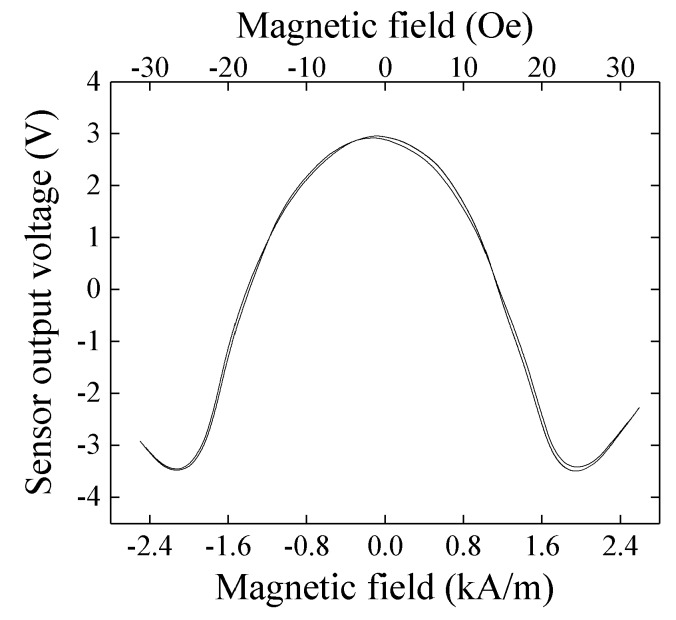
Measured result when a normal magnetic field B_Z_ = 0.11 T.

**Figure 9:**
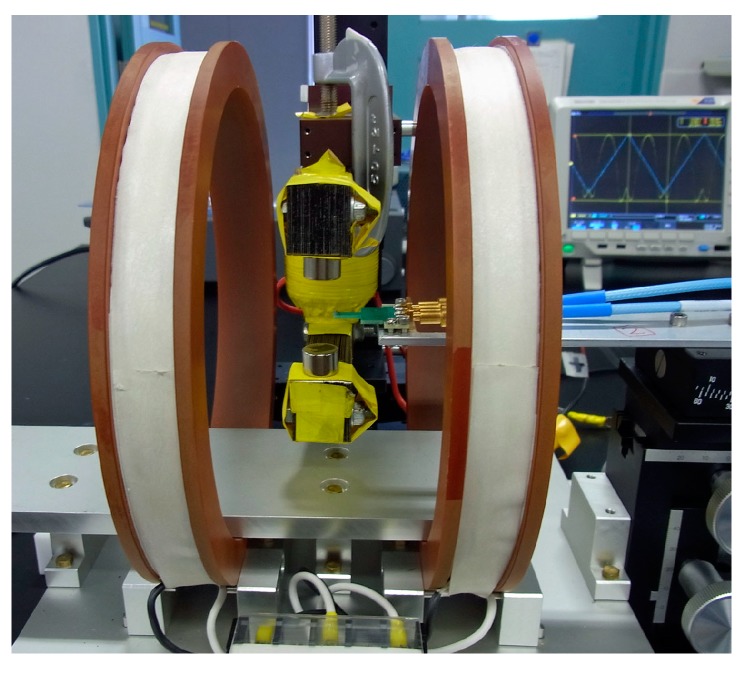
Photograph of the measurement apparatus with applying normal field.

Figure 8 shows measured results when a normal magnetic field, B_z_ = 1,100 G (0.11 T), was applied to the sensor element. In this measurement, the external magnetic field was applied in X direction. As the normal field increases, the vertical range of variation slightly decreases even it is keeping a low hysteresis of impedance variation. It means the sensitivity dV/dH does not decrease even in 0.11 T normal field, where V is the output voltage and H is the magnetic field. Here the normal field was applied by NdFeB magnets placed both on the upper side and on the lower side of the sensor element. These magnets were attached on an opening tip of a C-shaped magnetic core made by a bundle of silicone-steel sheets. The external field in the sensing direction was applied by a Helmholtz coil. A photograph of the measurement apparatus is shown in Figure 9. This result shows that the thin-film magneto-impedance sensor has a possibility to be useable even in hundreds of mT normal magnetic field and a strong candidate for detecting foreign inclusion in the manufacturing process.

**Figure 10:**
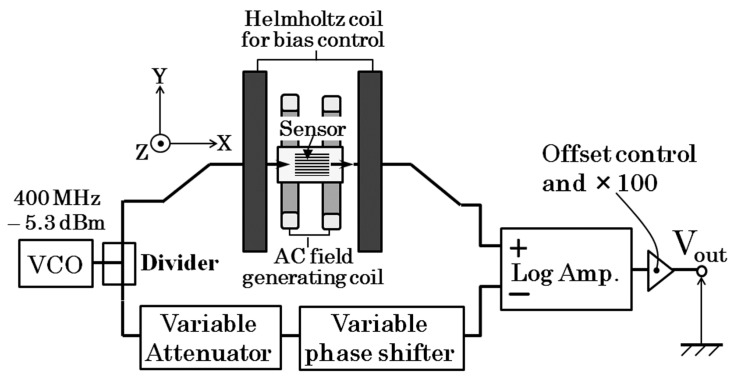
Measurement apparatus used in the sensitivity evaluation.

**Figure 11:**
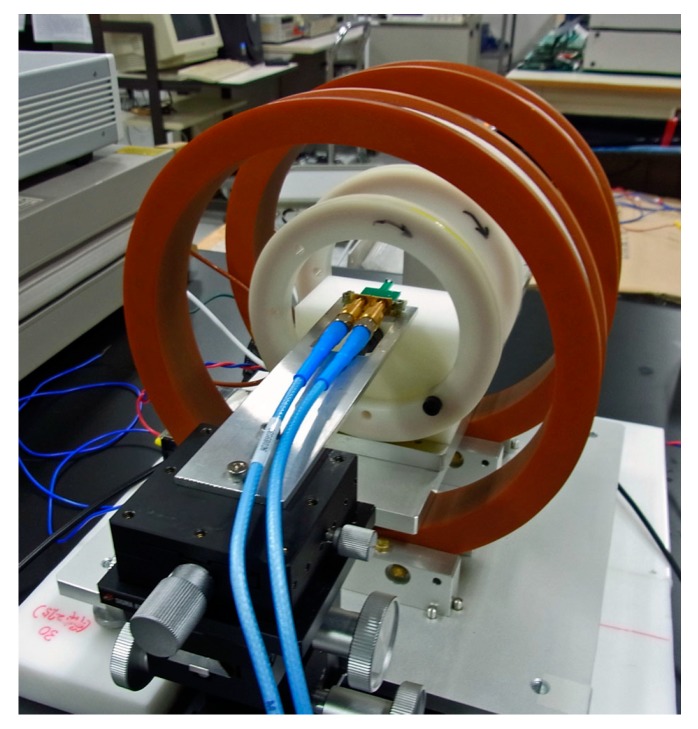
Photograph of dual-Helmholtz coil equipment used in sensitivity evaluation.

Now it proceeds to an evaluation of output property and sensitivity of the sensor system. This confirmation of sensing property was carried out without normal field. Figure 10 shows a schematic of measurement apparatus which was used in this experiment. Figure 11 shows a photograph of dual-Helmholtz coil equipment used in this study. One of the Helmholtz coil was used for applying a DC bias magnetic field and the other was used for generating a small AC magnetic field for the purpose of sensing limit evaluation. The biasing DC field was 1.4 kA/m (17.5 Oe) and the AC field was ranging from 0.5 × 10^−7^ T to 160 × 10^−7^ T. From here the applied magnetic field is assumed to be a value in vacuum, therefore it is expressed in a magnetic flux density in vacuum.

**Figure 12:**
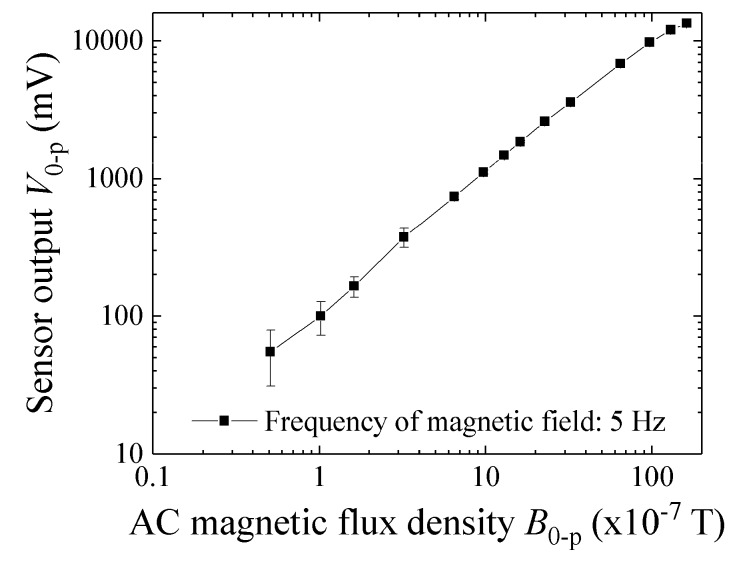
Variation of the sensor output as a function of AC magnetic flux density at 5 Hz.

Figure 12 shows a variation of the sensor output as a function of AC magnetic flux density at 5 Hz applied to the sensor element. This log-log plot shows that it has approximately linear relation. Due to a limit of output level of the final-stage amplifier, up to +14 V, the maximum value of output was +14 V in this study. The system developing in this study is designed for use in manufacturing process without magnetic shielding. Due to it a circumferential magnetic noise would be approximately several mG. With consideration of a background noise of the driving circuit, the minimum signal level must be larger than 0.1 mV in amplitude level. The output level of the developed driving circuit conforms to this design criterion. The AC field ranging from 1 × 10^−7^ T to 160 × 10^−7^ T corresponds to the output level ranging from 0.1 V to 14 V. 

The sensor sensitivity was measured by using spectrum analyzer. The sensitivity was defined as a minimum limit of the amplitude of magnetic field where a peak of the signal sunk under a noise level of the spectrum measurement. In this measurement, a DC-cut filter and a 20 dB attenuator was used for the reason of minimizing the noise level around DC and protect from over power. A Real Time Spectrum Analyzer (RTSA) was used for this measurement due to its ability of low frequency spectrum measurement including DC. The Tektronix RSA3408A was used for this measurement.

**Figure 13:**
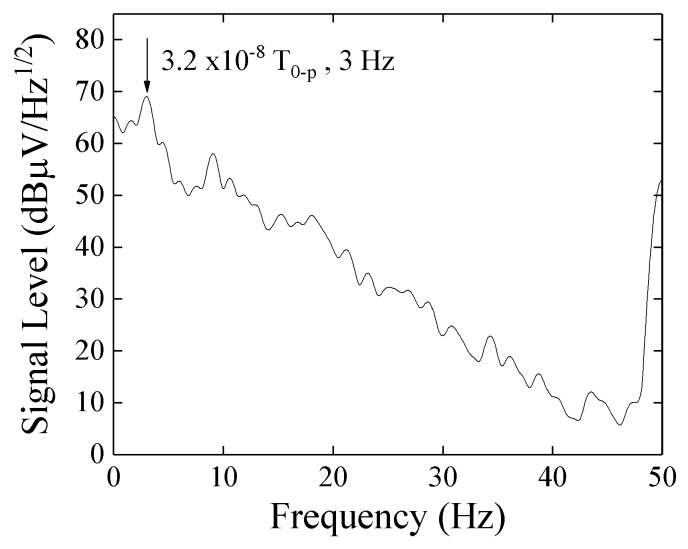
Result of spectrum measurement for a signal of 3.2 × 10^−8^ T_0-P_ at 3 Hz.

Figure 13 shows a measurement result. The horizontal-axis shows a frequency from DC to 50 Hz, the vertical-axis shows a signal level of output which is including alternating magnetic field, 3.2 × 10^−8^ T_0-P_ (0.32 mG_0-P_) at 3 Hz. From this figure, the 3 Hz peak clearly sticks out above the noise level. In other words, the sensitivity of the sensor system achieves 3.2 × 10^−8^ T (0.32 mG).

## 3. Discussion

A discussion on the experimentally obtained sensitivity using the proposed driving circuit and a future subject of this work would be carried out here. 

In this section, a cause of background noise and a relevance of achieved sensitivity are discussed.

**Figure 14:**
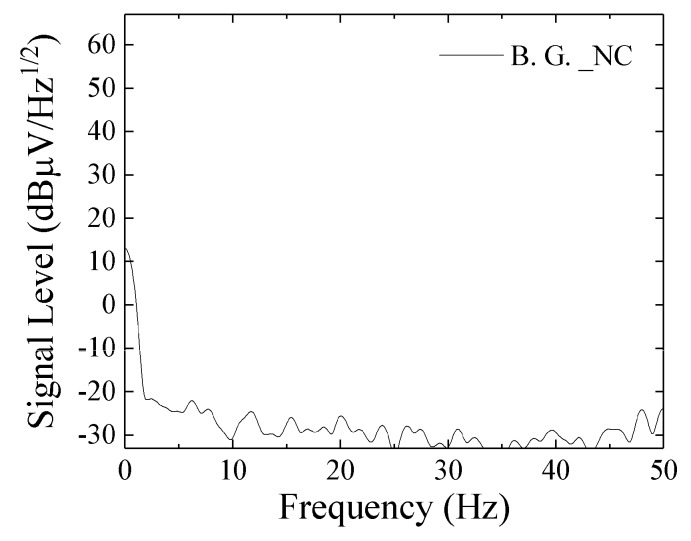
Noise level of measurement apparatus RSA3408A only.

**Figure 15:**
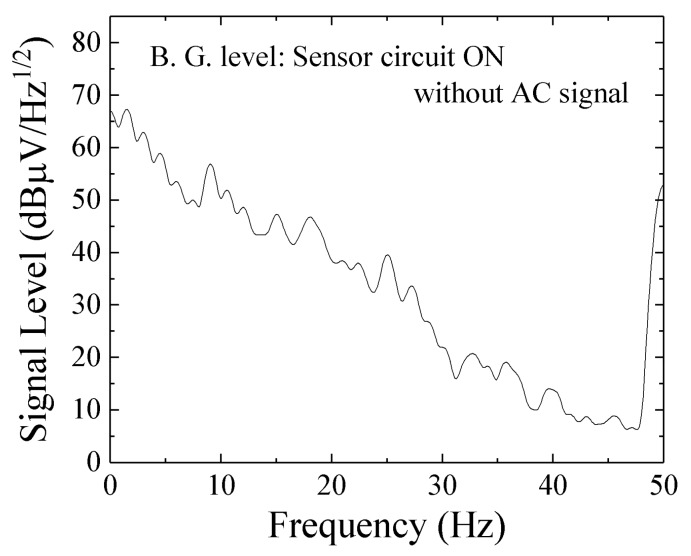
Noise level with connection of the sensor unit without AC magnetic field.

Figure 14 shows a noise level obtained for measurement apparatus RSA3408A only. The input was terminated by 50 Ω and the sensor system was not connected. Figure 15 shows a noise level with connection of the sensor unit without AC magnetic field. With comparison of them, the noise of measurement apparatus (RSA3408A, Tektronix) was lower than the one with sensor unit. The 50 Hz noise in Figure 13 and Figure 15 can be estimated as the Japanese commercial power supply signal. The 1/f-noise observed in the range from DC to 50 Hz would be estimated as a combination of system noise of the sensor driving circuit and circumferential magnetic field in the laboratory. The ratio of strength of these noises could be obtained by a measurement using magnetic shielding, but in my lab. we have not it.

**Figure 16:**
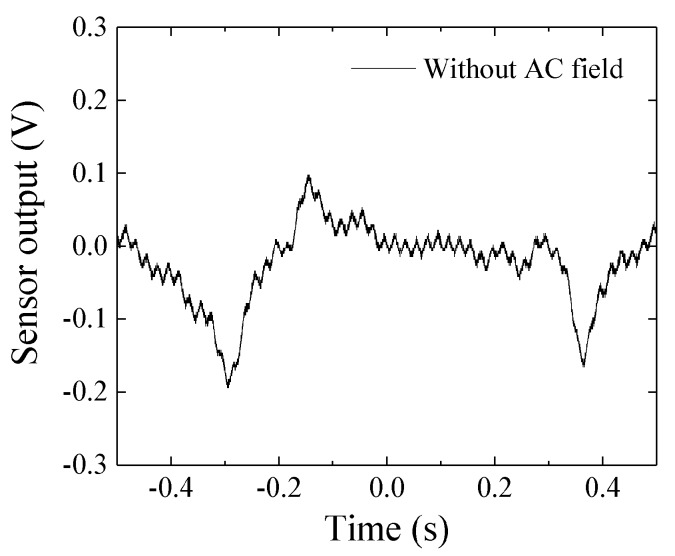
Measured time-domain noise of the sensor system.

Figure 16 shows an actual time-domain measurement result of the noise from the sensor system. This result was obtained using the sensor driving circuit without AC magnetic field. The 0.67 s interval dip-point was observed as a periodically repeated waveform with constant baseline, and it seems that it comes from the driving circuit. The small sine-signal is come from the commercial power supply because of the frequency 50 Hz. The previous report [7] was measured without using the electrically controlled attenuator and phase-shifter. It also used narrow band-pass filter at 300 Hz. If the cause of noise in the circuit will be cleared, the sensitivity will have a possibility to go up nearly 1.7 × 10^−9^ T.

**Figure 17:**
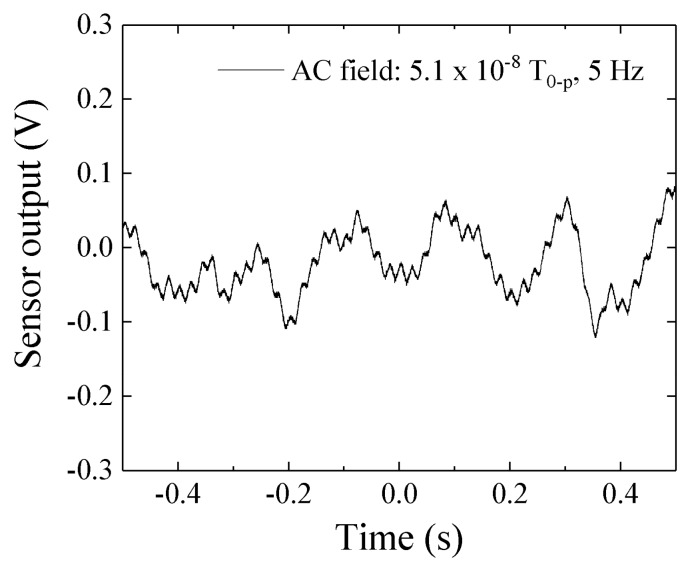
Measured signal when a 5.1 × 10^−8^ T_0-P_ in 5 Hz was applied to the sensor element.

Figure 17 shows a measured signal when a 5.1 × 10^−8^ T_0-P_ in 5 Hz was applied to the sensor element. We can see the periodical sine signal in this measurement. It is natural that this sine-signal is combined with the system-noise (Figure 16), but the 0.2 s periodical peak is clearly observed.

The achieved sensitivity 3.2 × 10^−8^ T_0-P_ shown in the results may be reasonable based on Figure 17. We can easily understand that a reduction of the system noise and 50 Hz noise could make the sensitivity drastically improve. An estimation of system sensitivity with application of the strong normal magnetic field is a future subject. The sensor-head apparatus shown in Figure 9 suffered a vibration coming from both the C-shaped core and the sensor fixing metal-arm. The more rigid apparatus and larger electro-magnetic core for generating a normal field will be needed for the precise experiment. I am now preparing such apparatus and will soon make a report about it.

## 4. Conclusion

A driving circuit for a thin-film magneto-impedance sensor using 400 MHz logarithmic amplifier is proposed. The highest sensitivity of the sensor used in this study is around 500 MHz, and the result of this article is well approaching and applicable to this frequency. An expected application of this sensor system is a nondestructive inspection of foreign matters in industrial products and detection of magnetic nanoparticles in drug solutions. For applying these targets, the frequency range of detection signal must be under several Hz. The proposed circuit using a logarithmic amplifier detects 400 MHz signal and make an output of the suitable frequency range. The achieved sensitivity of this system was 3.2 × 10^−8^ T at 3 Hz. This is a result without strong normal magnetic field, but it is expected to have nearly the same sensitivity in case of applying normal field because of the slight degradation of MI property of the sensor element (Figure 7). The sensitivity evaluation in a strong magnetic field will soon be reported in a future article.
